# Breast Milk MicroRNAs Related to Leptin and Adiponectin Function Can Be Modulated by Maternal Diet and Influence Offspring Phenotype in Rats

**DOI:** 10.3390/ijms23137237

**Published:** 2022-06-29

**Authors:** Marta Alonso-Bernáldez, Antoni Asensio, Andreu Palou-March, Juana Sánchez, Andreu Palou, Francisca Serra, Mariona Palou

**Affiliations:** 1 Alimentómica S.L. (Spin off no. 001 from UIB), Parc Bit, 07122 Palma de Mallorca, Spain; marta.alonso@alimentomica.com (M.A.-B.); antoni.asensio.torres@gmail.com (A.A.); apalou@alimentomica.com (A.P.-M.); mariona.palou@uib.cat (M.P.); 2 Laboratory of Molecular Biology, Nutrition and Biotechnology (Nutrigenomics, Biomarkers and Risk Evaluation Group), University of the Balearic Islands, 07121 Palma de Mallorca, Spain; joana.sanchez@uib.es (J.S.); andreu.palou@uib.es (A.P.); 3 Institut d’Investigació Sanitària Illes Balears (IdISBa), 07120 Palma de Mallorca, Spain; 4 CIBER de Fisiopatología de la Obesidad y Nutrición (CIBEROBN), Instituto de Salud Carlos III (ISCIII), 28029 Madrid, Spain

**Keywords:** milk-derived miRNAs, leptin/adiponectin ratio, metabolic programming, maternal diet, lactation

## Abstract

There is evidence of the role of milk components in the metabolic programming of offspring. Here, we aimed to investigate the effects of a diet during lactation on breast milk leptin, adiponectin, and related miRNAs’ expression, and their impact on dams and their offspring. Dams were fed a control diet (controls) or a diet enriched with oleic acid, betaine, and leucine (TX) throughout lactation. A TX diet promoted higher leptin at lactation day (LD) five and lower adiponectin on LD15 (vs. controls) in milk, resulting in increased leptin to adiponectin (L/A) ratio throughout lactation. Moreover, TX diet reduced milk levels of miR-27a, miR-103, miR-200a, and miR-222. Concerning TX offspring, higher body fat was early observed and maintained into adult life, accompanied by higher HOMA-IR than controls at three months of age. Offspring body fat content in adulthood correlated positively with milk L/A ratio at LD15 and negatively with miRNAs modulated by the TX diet. In conclusion, maternal diet during lactation can modulate leptin and adiponectin interplay with miRNAs in milk, setting up the metabolic programming of the offspring. Better knowledge about the influence of diet on this process is necessary to promote a healthy adult life in the progeny.

## 1. Introduction

Early postnatal life, particularly lactation, is a critical window of development, with a determinant role in the programming of later metabolic health [1,2]. Epidemiological evidence reports that breastfeeding protects against the development of obesity and related alterations compared to infant formula feeding [3,4]. In this sense, breast milk appears as a functional food for infants, providing nutrients and bioactive compounds at optimal concentrations during this period. Although the mechanisms responsible for the beneficial effects of breast milk remain uncertain, some studies point to specific milk compounds as major players, such as leptin and adiponectin [5,6]. In this line, leptin orally given during lactation to suckling rats has been described to exert protective effects on food intake and body weight control in adulthood [7,8,9]. Indeed, indirect evidence also exists in humans, showing an inverse correlation between breast milk leptin levels and body weight or body weight gain of lactating infants [10,11], supporting the idea of leptin as a critical molecule for correct metabolic programming and, thus, a healthy energy balance later in life [11]. Regarding adiponectin, contradictory data about its role in milk on the later metabolic health of the offspring has been previously published [12,13,14]. The interaction between leptin and adiponectin, generally referred to as the leptin to adiponectin (L/A) ratio, has been reported to be significant in metabolic processes [15], suggesting plasma L/A ratio as a marker of insulin resistance [16,17]. However, the relationship between the L/A ratio in milk and the adult metabolic health of infants has not been explored. Besides, we have previously shown that the breast milk supply of microRNAs (miRNA) related to leptin and adiponectin signalling in milk is dependent on maternal BMI, specifically that of miR-103 and miR-222 [18]. Another study of our group demonstrated how miR-26a, miR-27a, miR-200a, and miR-222 milk levels were affected by a maternal diet associated to hyperleptinemia [19]. In fact, these miRNAs have been involved in leptin modulation within the melanocortinergic system [20,21], such as the miR-200 family or in mammary metabolism, like miR-221 [22]. All in all, the levels of miR-26a, miR-27a, miR-103, miR-200a/b, miR-221, and miR-222 have been related to adipose tissue, mammary metabolism, and/or leptin and adiponectin signalling in milk [18,19,22,23]. Thus, these milk miRNAs were selected for this study, as they can act as modulators of L/A signalling, targeting gene expression in offspring tissues.

Maternal conditions can modulate breast milk composition, and the characteristics of maternal diet are a relevant factor [24]. Hence, different dietary patterns can modulate not only breast milk macronutrient composition but also the concentration of relevant bioactive compounds, such as miRNAs, together with other functional molecules like immunoglobulins [25]. Given the important role of bioactive compounds in milk composition, dietary strategies to modulate its concentration can be undertaken to facilitate the correct imprinting in the offspring. In this sense, three compounds have attracted our interest. Oleic acid (OA), a main ingredient in olive oil, contributes to the prevention of cardiovascular diseases [26,27] and, alongside its anti-inflammatory properties, various reports have stated its beneficial effects on lipid metabolism. The feasibility to provide physiological concentrations of OA in breast milk by modulating maternal dietary fat is of particular relevance [28]. Betaine is a non-essential nutrient present in different food sources, such as wheat or spinaches, that performs important physiological functions based on epigenetic mechanisms, and its dietary intake may contribute to supporting health status [29]. There is a health claim supporting the beneficial effect of betaine intake in maintaining normal homocysteine levels and heart health [30]. Moreover, together with this well-known role in cardiovascular function, betaine is also involved in lipid metabolism [31,32,33]. Lastly, leucine (Leu), an essential branched amino acid, has been shown to modulate lipid metabolism and energy homeostasis as well [34]. Previous studies in our group have shown some beneficial effects of maternal Leu supplementation during the suckling period in the later offspring life in rats, particularly in slimmer males [35,36]. Hence, these three nutrients are safely present in a healthy diet, and there is a rationale for their involvement in the modulation of milk composition and quality. Therefore, this study aimed to analyse the effect of a diet enriched with OA, betaine, and Leu (TX diet) in lactating rats on the modulation of milk composition, particularly milk miRNAs’ levels related to leptin and adiponectin signalling, and its potential short- and long-term impact in the dams and offspring phenotypes.

## 2. Results

### 2.1. Weight-Related Parameters and Food Intake of Dams

**Figure 1:**
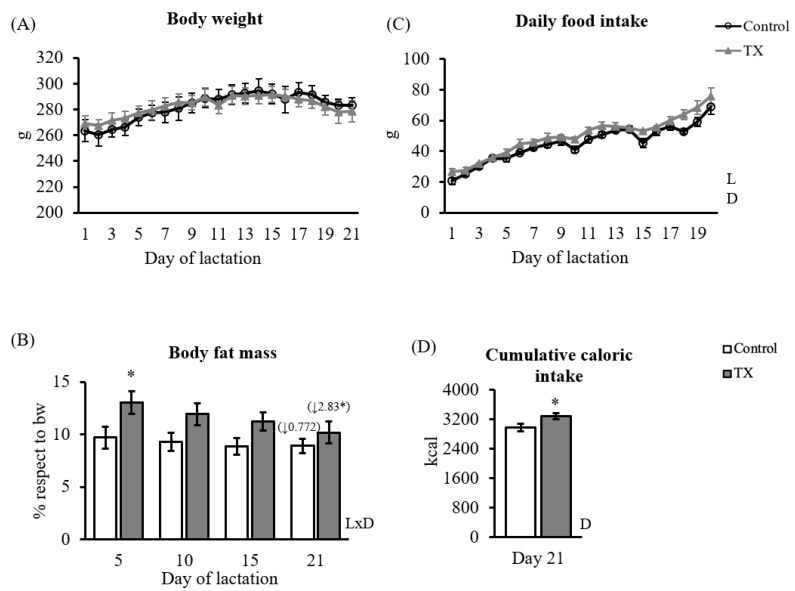
Body weight and intake data of dams throughout lactation: body weight evolution (**A**), body fat mass (**B**), daily intake evolution (**C**), and cumulative intake at the end of lactation (**D**). Data are mean ± SEM (*n* = 7). (**B**) The percentage of body fat decrease throughout lactation is indicated in brackets with a down arrow for each group. Statistics: A repeated measures ANOVA was carried out to study the effect of maternal diet and day of lactation: LxD, the interaction between lactation day and TX diet. Single comparisons between groups were performed by Student’s *t*-test: *, different from controls. Threshold significance was set at *p* ≤ 0.05 for all tests.

The body weight, food intake, and body fat mass of the dams are presented in Figure 1. Body weight evolved in a similar way between experimental groups throughout lactation (Figure 1A), but no significant differences were observed among them. TX dams showed a tendency to present higher body fat mass than controls, which was only significant on day 5. TX dams showed higher daily food intake and cumulative caloric intake from day one to the end of lactation compared to controls. Interestingly, the decrease in body fat content between days 5 and 21 of lactation was greater in TX dams compared to controls. At the time of sacrifice, day 21 of lactation, no significant differences were observed in blood glucose or tissue weight between groups.

### 2.2. Leptin and Adiponectin in Dams

**Figure 2:**
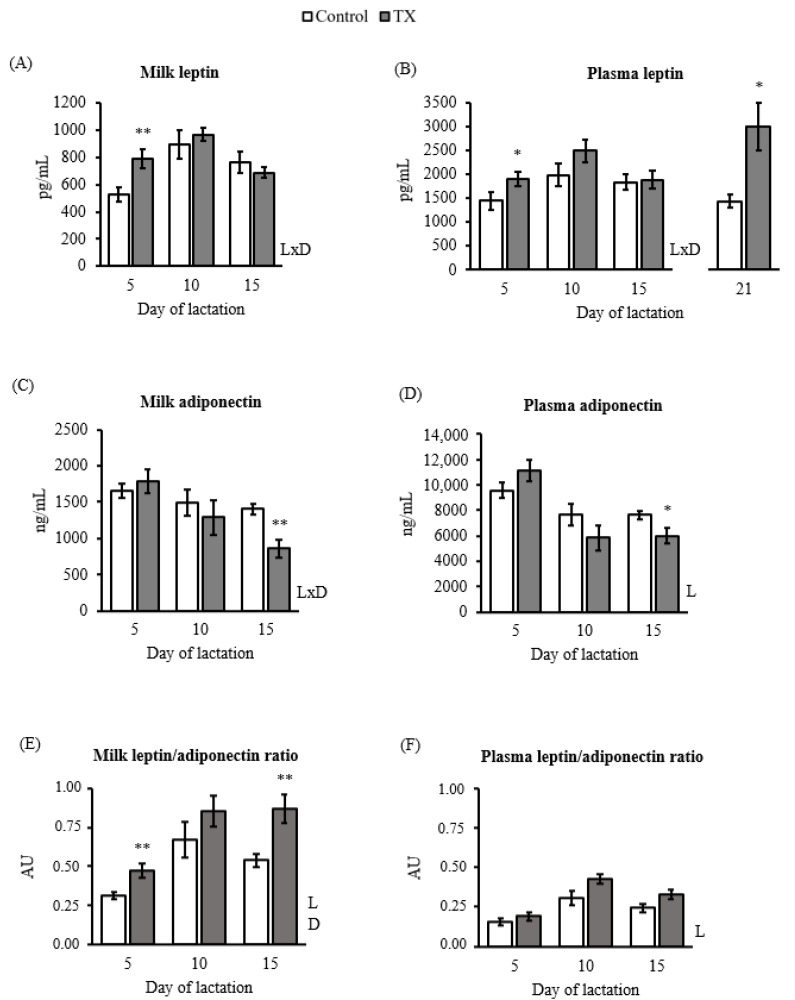
Leptin and adiponectin levels throughout lactation. Leptin concentration during lactation in milk (**A**) and plasma was obtained from a saphenous vein (**B**) (on day 5, 10, and 15 of lactation) and trunkal blood (on day 21). Adiponectin levels at days 5, 10, and 15 of the suckling period in milk (**C**) and plasma (**D**) were obtained from the saphenous vein. The Leptin/Adiponectin (L/A) ratio in milk (**E**) and plasma (**F**). Data are mean ± SEM (*n* = 7). Statistics: A repeated measures ANOVA was carried out to study the effect of maternal diet and day of lactation: L, effect of the lactation day; D, the effect of TX diet; and LxD interaction between lactation day and TX diet. Single comparisons between groups were performed by Student’s *t*-test: *, different from controls. Threshold significance was set at *p* ≤ 0.05 for all tests (**, *p* ≤ 0.00).

Leptin and adiponectin levels in milk and plasma, as well as the L/A ratio, of TX and control dams are shown in Figure 2.

The TX group presented higher leptin concentration in milk and plasma on day 5 of lactation (Figure 2A,B) showing an increase of nearly 50% and 32%, respectively, compared to controls. No significant differences were observed on day 10 or 15 of lactation. Regarding plasma, at the end of lactation (day 21), circulating leptin levels were significantly higher in the TX group compared to control rats. Of note, there was a significant interaction between lactation day and TX diet in both milk and plasma leptin levels.

Concerning adiponectin (Figure 2C,D), TX rats presented significantly lower levels in milk and plasma on day 15 of lactation, but not previously, compared to the controls. Like the leptin pattern, an interaction between the day of lactation and the TX diet was also observed in milk adiponectin.

L/A ratio in milk was significantly increased in TX dams compared to controls, being more evident at days 5 (↑23%) and 15 (↑32%) of lactation. Both milk and plasma L/A ratios were modulated by the day of lactation. No significant differences were found in the plasma L/A ratio associated with diet along with lactation.

### 2.3. Breast Milk miRNAs

**Figure 3:**
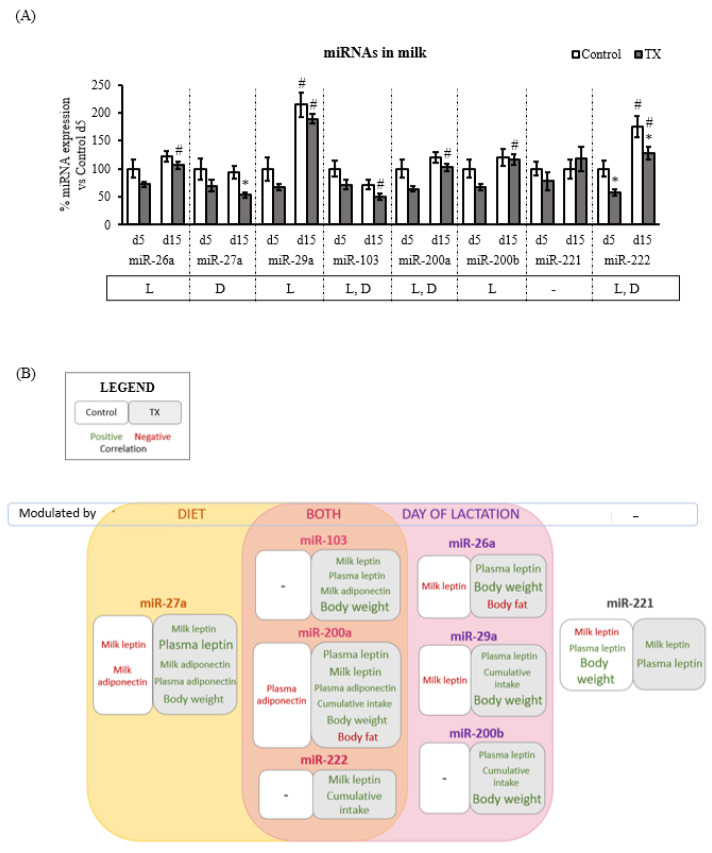
Levels in the milk of several miRNAs involved in leptin signalling in the control and TX groups at days 5 and 15 of lactation (**A**) and a schematic representation of the associations found between these miRNAs and milk leptin and adiponectin and other phenotypical variables (**B**). (**A**) Results are expressed relative to the miRNA expression values at day 5 of controls for each miRNA. Data are mean ± S.E.M. (*n* = 7). (**B**) In yellow are the miRNAs exclusively modulated by maternal diet. Those that were modulated by the day of lactation are shown in purple, and those that were modulated by both factors are shown in pink. The correlations found in the control group are shown in the white boxes, whereas the ones observed in the TX dams are grouped in grey. If the variable is represented in green, it means a positive correlation; otherwise, it is represented in red. The larger the variable represented, the stronger the correlation throughout lactation time points. Statistics: repeated measures ANOVA was carried out to study the effect of maternal diet and day of lactation: L, effect of the lactation day; D, the effect of TX diet. Single comparisons between groups were performed by Student’s *t*-test: *, different from controls at same time point; # different from their group at day 5. To study the associations between milk miRNAs and variables of interest Spearman correlation analysis was performed. Threshold significance was set at *p* ≤ 0.05 for all tests.

Eight miRNAs potentially related to leptin signalling were selected based on previous results (miR-26a, miR-27a, miR-29a, miR-103, miR-200a, miR-200b, miR-221, and miR-222). Determinations were performed in milk samples at two representative time points of lactation (days 5 and 15) (Figure 3A). Along with lactation, the following breast milk miRNAs were increased: miR-26a, miR-29a, miR-200a, miR-200b, and miR-222. This pattern could be considered specific, as miR-27a and miR-221 concentrations were found stable between days 5 and 15. A decrease was observed in miR-103 during this period. Concerning the impact of the diet, the TX combination promoted a general tendency to decrease the milk miRNAs analysed. Specifically, significantly lower levels were observed for miR-27a (≈↓37%), miR-103 (≈↓30%), miR-200a (≈↓26%), and miR-222 (≈↓35%).

### 2.4. Milk miRNAs and Phenotypical Variables Interplay

Spearman’s correlation analysis between phenotypical variables, including leptin and adiponectin concentrations, and milk miRNAs was performed to obtain an overview of the complexity of interrelationships associated with the dietary treatment. A schematic representation summarizing the results is displayed in Figure 3B, and the whole set of data with the milk miRNAs’ levels on days 5 and 15 are presented in Table S2 and Table S3, respectively.

Milk leptin was negatively correlated with miR-26a, miR-27a, miR-29a, and miR-221 at day 5 in the control group. No correlations were observed concerning miRNAs and plasma leptin in the control dams. In contrast, the TX diet promoted a distinct, inverse, and stronger pattern between leptin and its modulation by miRNAs. Positive correlations were found involving leptin, specifically with miR-27a, miR-103, miR-200a, and miR-221 in both milk and plasma; with miR-221 and miR-222 in milk; and with miR-26a, miR-29a, and miR-200b in plasma (Figure 3B).

Similarly, in the control group, adiponectin negatively correlated at day 15 with miR-27a in milk and with miR-200a in plasma. However, positive correlations were found between adiponectin and miRNAs on day 5 associated with the TX diet. Specifically, adiponectin positively correlated with miR-27a in both milk and plasma, as well as with miR-103 in milk and miR-200a in plasma in the TX group.

Finally, a differential impact on the relationship between milk miRNAs and body weight was also observed. In control animals, body weight was positively associated only with milk miR-221 (at day 15), the only one that was not modulated by the lactation period or diet. In contrast, in the TX group, body weight positively correlated throughout lactation with most milk miRNAs (miR-26a, miR-27a, miR-29a, miR-103, miR-200a, and miR-200b at day 5) except for miR-221 and miR-222. Additionally, in TX rats, body fat negatively correlated with miR-26a and miR-200a at day 15, while the cumulative intake was strongly associated with miR-222 throughout lactation and, to a minor extent, with miR-29a, miR-200a, and miR200b in the TX treatment.

### 2.5. Tissue Gene Expression in Dams

To further characterize the effect of the TX diet, the expression of genes codifying for leptin, adiponectin, and their receptors, as well as relevant genes in lipid metabolism, were analysed in key tissues: mammary gland (MG), liver, retroperitoneal white adipose tissue (rWAT) and brown adipose tissue (BAT), at the end of lactation (day 21).

**Figure 4:**
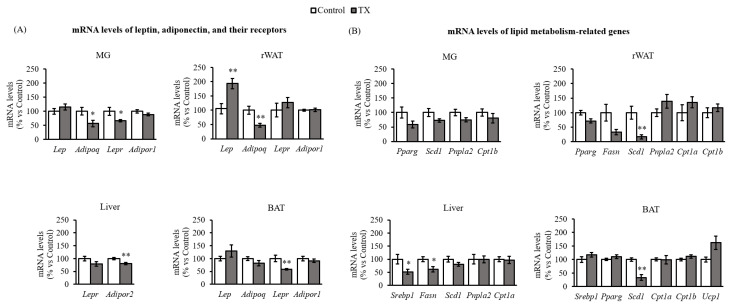
mRNA levels of leptin, adiponectin, and their receptors (**A**), and lipid metabolism-related genes (**B**) in MG, rWAT, liver, and BAT at the end of lactation. Data are mean ± SEM (*n* = 7). Results are expressed relative to the gene expression value of the controls for each gene. Single comparisons between groups were performed by Student’s *t*-test: *, different from controls. Threshold significance was set at *p* ≤ 0.05 (**, *p* ≤ 0.00). Abbreviations: leptin (*Lep*), adiponectin (*Adipoq*), leptin receptor (*Lepr*), adiponectin receptor *(Adipor*), peroxisome proliferator activated receptor gamma (*Pparg*), stearoyl-CoA desaturase-1 (*Scd1*), patatin-like phospholipase domain-containing protein 2 (*Pnpla2*), carnitine palmitoyltransferase 1 (*Cpt1*), fatty acid synthase (*Fasn*), sterol regulatory element binding protein 1 (*Srebp1*), uncoupling protein 1 (*Ucp1*).

Notably, TX dams showed a twofold induction of leptin (*Lep*) mRNA (≈90%) in rWAT compared with controls, but no differences were observed in other tissues (MG or liver) (Figure 4A). Lower mRNA expression of the gene coding for adiponectin (*Adipoq*) was observed in MG (↓43%) and rWAT (↓53%) in TX dams in comparison to the controls, and no differences were observed in the expression in BAT. Finally, expression of the respective receptors was also diminished in a tissue-specific manner. Lower expression of *Lepr* in MG (↓33%) and BAT (↓42%) and of *Adipor2* in the liver (↓20%) was shown.

Regarding lipid metabolism (Figure 4B), a general trend of diminished lipogenesis capacity in the tissues studied was found in the TX group. Particularly, in TX dams, *Scd1* mRNA expression was acutely reduced in rWAT and BAT by 83 and 66%, respectively, while *Fasn* mRNA levels were lower in the liver (↓38%) and rWAT (↓67%, *p* = 0.060) compared to controls. These results were consistent with the lower expression of the lipogenesis-related transcription factor *Srebp1* in the liver (↓48%) and the observed tendency towards an increase in *Ucp1* mRNA levels in BAT (*p* = 0.061), which altogether might help explain the changes in TX dams’ body composition.

### 2.6. Offspring Characterization

**Figure 5:**
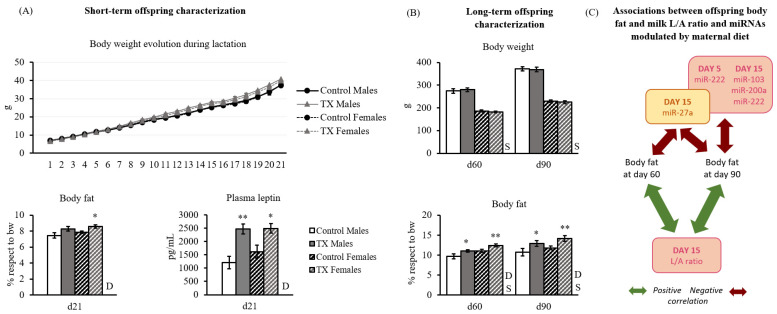
Offspring characterization in the short term (**A**) and long term (**B**). (**A**) Body weight evolution was tracked throughout the lactation period, and on day 21, body fat and plasma leptin were measured (*n* ≈ 35). (**B**) Body weight and body fat were measured at days 60 and 90 of age (*n* = 16). (**C**) Schematic representation of the associations observed between offspring body fat mass and milk L/A ratio and the miRNAs modulated by maternal diet. In yellow is the variable exclusively modulated by maternal diet, and in pink, those that were modulated by both maternal diet and day of lactation. Positive correlations are represented with green arrows, whereas the negatives are in red. Data are mean ± SEM. Statistics: A two-way ANOVA was carried out to study the effect of maternal diet and sex: D, the effect of maternal TX diet; S, sex effect. Single comparisons between groups were performed by Student’s *t*-test: *, different from their respective control. To study the associations between offspring body fat and milk L/A and diet-modulated miRNAs, Spearman correlation analysis was performed. Threshold significance was set at *p* ≤ 0.05 for all tests (**, *p* ≤ 0.00).

During lactation, no differences were observed in body weight evolution associated with the maternal treatment (Figure 5A). However, offspring born to dams fed TX diet during lactation presented higher plasma leptin and percentage of body fat compared to their controls at the end of lactation (Figure 5A). The offspring followed the same pattern up to three months of age, with no differences in body weight among groups, but higher body fat in the adult TX progeny (Figure 5B). No significant differences were found in cumulative food intake due to maternal diet (data not shown). To assess whether offspring body fat in adulthood could be related to breast milk characteristics, associations between the percentage of body fat mass (at days 60 and 90), milk L/A ratio, and diet-modulated miRNA were assessed by Spearman’s correlation analysis (Table S4). Interestingly, female offspring’s body fat mass in adulthood positively correlated with the L/A ratio in milk at day 15 of lactation at both ages. In males, this same tendency was observed, although not significant. Regarding milk miRNAs, their levels were associated with body fat in adulthood. Milk miR-27a, miR-103, miR-200a, and miR-222 at day 15 negatively correlated with offspring fat mass at day 90. This association was already observed at day 60 in the case of miR-27a in females, while in males, miR-222 levels at day 5 already correlated with body fat mass at day 90. A schematic representation of these results is displayed in Figure 5C.

**Figure 6:**
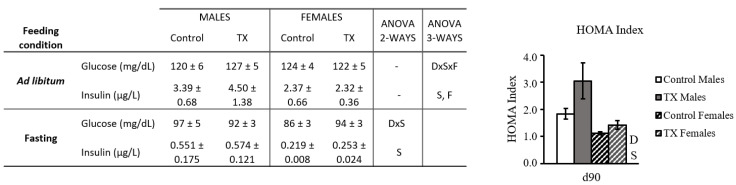
Long-term offspring glucose and insulin characterization. Insulin and glucose levels are presented in the table and HOMA index in the graph to the side. Data are mean ± SEM (*n* = 8). Statistics: two-way ANOVA was performed to study the effect of maternal diet and sex. Then, a three-way ANOVA was carried out to also study the effect of feeding conditions: D, the effect of maternal TX diet; S, sex effect; F, feeding conditions effect. Threshold significance was set at *p* ≤ 0.05.

Circulating glucose and insulin under fed and fasting conditions and HOMA-IR index at the age of 90 days are shown in Figure 6. Notably, the HOMA index was significantly higher in the TX group compared to the control one (*p* ≤ 0.05, two-way ANOVA).

## 3. Discussion

Perinatal nutrition, particularly during lactation, is a key programming factor of future health. Specific bioactive compounds naturally present in breast milk, such as leptin, are provided to the neonate and contribute to their metabolic programming [11]. Additionally, miRNAs related to leptin and adiponectin signalling are present in breast milk, and their levels depend on maternal BMI [18]. We have shown that breast milk miRNAs can modify the expression of target genes and exert direct or indirect programming effects in offspring [19,23]. However, little knowledge exists on the associated impact of maternal dietary components on these modulators, the quality of breast milk, or their long-term impact on the offspring. Here, we provide evidence that an enriched diet with OA, betaine, and Leu, supplied during lactation, was able to promote increased leptin and adiponectin concentrations in rats’ breast milk. Lactation was accompanied by increased expression of specific milk-derived miRNAs, and the supplementation counteracted the extent of this increase. Therefore, lower expression of some milk miRNAs was associated with the supplementation and resulted in offspring metabolic traits observed at an early stage and remaining throughout later life.

The formulation tested in this research was based on published data providing evidence for the potential benefits that single compounds exert against obesity, particularly during the perinatal period, and on own preliminary results showing the potential effect of the triple combination in a primary culture of rat mammary epithelial organoids. To our knowledge, this is the first time this enriched combination of nutrients has been administered during lactation. Only a few studies were found using individual supplementation of these compounds (or foods containing them) and exploring the dams’ metabolic response and offspring programming of later metabolic health. They have provided the rationale for the formulation design. In brief, rat dietary supplementation during pregnancy and lactation with OA does not affect body weight or food intake of dams, but the offspring show lower body weight at weaning than the controls, although the differences disappear in adulthood. Interestingly, OA milk levels negatively correlate with pups’ body weight gain and positively with UCP1 content in offspring BAT, suggesting that maternal OA consumption stimulates BAT thermogenic capacity in suckling pups [28,37]. The TX formulation, although containing a lower amount of OA, was mirroring, to some extent, the impact on the BAT thermogenic potential, as the expression of *Ucp1* in dams was induced (↑62%), although it did not attain statistical significance. Concerning the potential impact of higher consumption of OA, it is interesting to mention the results found regarding the Mediterranean diet (MD) intake, given its characteristic contribution of olive oil, unlike other diets. It has been shown that adherence to MD is associated with lower fat mass in breastfeeding mothers [38] and can impact plasma levels of miRNAs related to metabolic syndrome [39]. However, little is known about the influence of this diet on milk bioactive compounds and metabolic programming.

Dietary betaine is the subject of several studies because of its function as a methyl-donor group and its beneficial effects on oxidative stress, metabolic inflammation, and the prevention of fatty liver development [40,41]. Specifically, betaine is involved in the regulation of lipid and energy metabolism. Both in vitro cell studies and animal models have contributed to showing that betaine inhibits hepatic fat accumulation and promotes mitochondrial performance by impairing fatty acid synthesis and promoting its oxidation and secretion [40,42,43,44]. A meta-analysis of RCT human studies also confirms this role, as betaine supplementation (2–6 g/day) reduces body fat mass, although it is associated with minor effects on body weight or BMI [45]. According to the role of betaine on related-epigenetic mechanisms, maternal betaine supplementation has also been characterized in animal models during the perinatal period. Supplementation with betaine during rat pregnancy and lactation (1%) decreases the dexamethasone-induced fatty liver in adulthood in female offspring mediated by the downregulation of the expression of lipogenic-related genes [46]. Similarly, dietary betaine supplementation during sow pregnancy (3 g/kg) reduces hepatic lipogenesis in neonatal piglets [47]. Our data on dams’ gene expression was fitted with a metabolic profile with diminished lipogenic potential in the key tissues (liver and adipose). However, the induction of lipid mitochondrial oxidative capacity or the reduction in body fat could not be observed. Both dams and offspring showed higher body fat content during the study. In dams, this was only observed after five days of supplementation (Figure 1B), and in offspring, it was observed at weaning and later. In any case, the daily dose of betaine supplied by the TX diet (1.6 g/kg) during lactation was lower than the doses reported in pregnant animals [46,47]. Therefore, the TX formulation replicated some of the beneficial effects seen on lipid metabolism based on related gene expression, although its impact on reducing body fat was minimized.

Concerning the Leu content in the TX formulation, previous data of the group revealed that dietary Leu supplementation (2%) to lactating dams does not affect their body weight, daily food intake, or thermogenesis. However, dams tend to have a higher lean/fat ratio throughout lactation, which is associated with lower orexigenic neuropeptide expression in the hypothalamus [35]. Interestingly, a differential gender-associated imprinting is related to maternal Leu supplementation during lactation, as Leu exerts some protection against the development of obesity in adult male offspring. However, in contrast, it is associated with a greater predisposition to diet-induced obesity in adult female offspring. Neuronal architecture in the hypothalamus, involving neuropeptide Y (NPY) fibres and expression of neuropeptides and factors of the mTOR signalling pathway, takes part in the mechanisms protecting the males and predisposing Leu-females to obesity under the appropriate environment [36]. In TX formulation, the Leu content provided with diet was lower, which may have contributed to minimising its specific impact on progeny.

In summary, the formulation was designed to test a supplementation with moderate doses of the nutrients of interest to induce the beneficial effects described individually, particularly on body composition and lipid metabolism. The results confirmed the induction of specific metabolic adaptations exerted by the maternal TX diet, through milk bioactive compound supply, on the offspring phenotype, such as leptin, adiponectin, and miRNAs.

Breast milk leptin and adiponectin concentrations showed opposite patterns along with lactation. On the one hand, an increment of leptin concentration at the first stages of lactation but diminished adiponectin levels later were observed in TX dams’ milk. This resulted in a significant increase in the milk L/A ratio of TX dams compared to their controls throughout lactation. On the other hand, *Adipoq* expression was decreased in TX dams in both rWAT and MG compared to controls. The changes in the production and levels of leptin and adiponectin due to the enriched diet were accompanied by a reduction in the capacity of TX dams to respond to these hormones. In concrete, TX dams showed lower *Lepr* gene expression in both BAT and MG, besides *Adipor2* mRNA levels decreased in the liver compared to control rats.

In vitro and in vivo evidence with individual treatments of OA, betaine. or Leu can at least partly shed light on the changes in leptin and adiponectin observed here. In vitro, OA, and Leu treatments to adipocytes resulted in increased leptin mRNA levels [48] and leptin secretion [49], suggesting a stimulatory effect of both compounds. Contrarily, betaine seems to have an inhibitory effect on leptin expression as its supplementation in mice reduces the circulating leptin concentration and the detrimental effects of an obesogenic diet [33]. Furthermore, in vitro treatment of betaine (250 µM, for 24 h) reduces mRNA expression of leptin in cultured human adipocytes [50]. Therefore, the impact of the TX diet concerning leptin modulation would be mainly ascribed to the combined role of OA and Leu as that of betaine was shown minimized. The TX diet resulted in incremented plasma leptin levels along with lactation and was associated with higher gene expression in rWAT at the end of the treatment. Concerning adiponectin, OA has been shown as a clear inhibitor of adiponectin production. Human adipocytes pre-treated with OA (1–100 μmol/L) before stimulation with TNFα show the total suppression of adiponectin secretion induced by TNFα treatment, as well as a reduction in *Adipoq* mRNA levels (compared with TNFα treated cells) [51]. Meanwhile, Leu and betaine supplementation has been shown to increase adiponectin. For instance, in vitro, a 4 mM Leu treatment of 24 h significantly increases protein expression levels of adiponectin and its receptor in porcine skeletal muscle cells [52]. However, in vivo, a 5% Leu supplementation after 15 weeks in high-fat diet rats raises serum adiponectin [53]. Regarding betaine, Wang et al. [33] displayed how a 1% (wt/vol) betaine supplementation for 12 weeks improves circulating adiponectin in mice fed a high-fat diet, which they later supported in primary adipocytes of these animals, in which treatment with different doses of betaine (0–1 mM) for 20 h shows an adiponectin increase in a dose-dependent manner. The fact that the doses in the TX diet were lower could explain the minimization of these compounds’ effect on adiponectin, pointing out OA as the main driver for adiponectin modulation. Overall, the TX formulation was able to replicate previously described metabolic features on adipokines’ changes. Although the participation of other factors cannot be ruled out, results showed metabolic adaptations associated with the specific intake of OA and Leu in lactating dams, whereas the influence of betaine was less noticeable.

The modulation of leptin/adiponectin in breast milk by promoting changes in the maternal dietary pattern can be of interest in preventing obesity in offspring. Previous investigations in animal models and humans support the significant relationship between breast milk leptin and adiponectin concentrations and infant body composition. Notably, the beneficial effects of an adequate intake of leptin during lactation in the programming of better control of energy balance seem to be clear, especially in children from normal-weight mothers [11]. Direct evidence from animal studies shows that leptin supplementation at physiological doses during the suckling period protects against overweight and related metabolic alterations in adulthood [8,9]. Meanwhile, breast milk adiponectin concentrations vary considerably across the lactational stage with high interindividual variation [54], and its exact role in infant growth and development remains controversial. It seems that breast milk adiponectin exhibits pleiotropic effects on the infant based on food availability, stage of life, and maternal BMI [54]. In this study, we showed that maternal diet modulated milk adipokines and that the consequent change in milk L/A ratio, and not leptin or adiponectin absolute levels *per se*, was associated with offspring phenotype. Notably, the milk L/A ratio at day 15 of lactation positively correlated with offspring body fat in adulthood. The fact that the milk L/A ratio was associated only at day 15 with offspring adult accumulation suggested a potential determinant role of the decreased levels of adiponectin in milk at this stage of lactation rather than the increment of leptin at the first stages of lactation. Our results pointed out the milk L/A ratio during the postnatal period as a potential indicator of newborns’ future metabolism and health.

To better comprehend milk adipokines’ modulation, the study of milk miRNAs appears of great interest. It has been proposed that miRNAs in milk could be absorbed by intestinal cells due to their high stability, mainly because of their encapsulation in exosomes and extracellular vesicles [55,56,57]. This safe delivery of miRNAs can exert biological functions and impact infant development [18,23,58,59]. Here we observed how a maternal diet able to modulate leptin and adiponectin levels in milk also altered the expression levels of miR-27a, miR-103, miR-200a, and miR-222. The fact that these miRNAs’ levels were significantly affected by the TX diet, whose dams presented a higher milk L/A ratio, supported the idea of a potential interplay between these miRNAs and the L/A axis, as proposed by other authors [18,19]. It has been previously reported that miR-27a, miR-103, and miR-222 levels are associated negatively with leptin concentration in breast milk in the second month of lactation but only in normal-weight mothers [18]. This is of relevance since overweight/obese mothers present higher milk leptin concentration compared to normal-weight mothers, which suggests an interplay between these miRNA expressions and leptin in milk. Although we did not observe significant differences in maternal body weight between animal groups, milk miR-27a levels at day 5 positively correlated with milk and plasma leptin throughout lactation in TX dams, which presented a higher L/A ratio, but not in controls. Furthermore, the miR-27a concentration on day 5 of lactation also correlated positively with the levels of plasma (day 10) and milk (day 5) adiponectin in TX rats but not in control rats. These results might suggest that levels of miR-27a in milk could reflect lactating leptin and adiponectin fluctuations at early stages in response to maternal diet, either by direct or indirect action. Hence, this miRNA appeared as a potential biomarker of the supply of leptin and adiponectin through breast milk to offspring, which, in turn, could envisage their programming and future metabolism, especially since levels of miR-27a did not change with the day of lactation. This TX-modulated miRNA was the one negatively associated with offspring body fat at both time points in adulthood. Moreover, these changes in the levels of specific breast milk miRNA after the TX diet can affect the offspring’s gene expression modulation, which cannot be ruled out as additional programming factors of later phenotypes. However, to demonstrate this cause—effect relationship, specific analyses are needed.

## 4. Materials and Methods

### 4.1. Animals and Experimental Design

Virgin female Wistar rats aged 9 months old were mated with male Wistar rats and housed independently under controlled temperature (22 °C), a 12-h light-dark cycle (lights on from 08.00 to 20.00 h), and unlimited access to tap water throughout the whole experiment. The following day after the pups were born (birthdate was set as day 0), dams were separated into two dietary groups and fed ad libitum during the whole lactation (days 1 to 21): Control group (*n* = 7), fed a standard chow diet; and TX group (*n* = 7), fed the TX diet, enriched with OA, Leu, and betaine. Diets were isocaloric and were supplied by SAFE diets (Augy, France). Their composition is summarized in Table 1.

### 4.2. Maternal Follow-Up

On day 1 of lactation rats were weighed and each litter was adjusted to ten pups per dam (five males and five females, when possible). Regarding dams, a daily record of food intake and body weight was carried out during the 21 days of lactation. On days 5, 10, and 15, milk and blood samples were collected under feeding conditions, and body fat was determined (by EchoMRI-700TM, Echo Medical Systems, LLC., Houston, TX, USA). This equipment conducts body composition scanning using quantitative magnetic resonance. For milk collection, 0.4 UI-kg of body weight of oxytocin (Falcilpart, Laboratory syva s.a.u; León, Spain) was administered intraperitoneally to favour milk production. After a few minutes, dams were anaesthetized by inhalation with isoflurane (IsoFlo, Abbott Laboratories Ltd., Chicago, IL, USA) to facilitate milk collection from mammary glands. Milk samples were extracted by manual milking from all teats, mixed, and stored at −80 °C until further analysis. Blood samples were extracted from the saphenous vein without anaesthesia into heparinized containers, and plasma was obtained by centrifugation at 700× *g* for 10 min and stored at 20 °C.

On day 21 (weaning), body fat was measured, and dams were sacrificed by decapitation during the first 2 h after the beginning of the light cycle and under feeding conditions. For each animal, trunk blood samples were obtained, and glucose concentration was determined. Retroperitoneal WAT (rWAT), brown adipose tissue (BAT), liver and mammary gland (MG) tissue were collected from dams. All tissues were rapidly removed, frozen in liquid N_2_, and stored at −80 °C until further study. All blood samples were centrifuged and stored as mentioned above.

### 4.3. Offspring Follow up

The body weight of the pups from control and TX dams was followed from day 1 to day 90 of age. At weaning (day 21) body fat mass was measured and blood was collected from a set of pups (1–2 males and 1–2 females from each dam) under feeding conditions. In addition, a set of pups (*n* = 16 animals per group and gender) was maintained alive in standard conditions (chow diet with free water access) and caged separately by sex and group, two per cage. Body weight and food intake (from weaning) were recorded until day 90, and body composition was determined at 2 and 3 months of age. To determine insulin sensitivity at 3 months of age, blood samples were collected in a set of animals under feeding conditions (*n* = 8) and in another set under 12-h fasting conditions (*n* = 8), following the same procedure as described above.

### 4.4. Analytical Determinations in Biological Fluids

Glucose levels were measured in fresh blood with an Accu-Check Glucometer (Roche Diagnostics, Barcelona, Spain).

Leptin and adiponectin concentrations were determined in plasma and milk using a mouse/rat leptin and a rat adiponectin ELISA kit (R&D Systems, Minneapolis, MN, USA); circulating insulin was determined with a commercial rat insulin ELISA kit (Mercodia, Sylveniusgatan, Sweden). In all cases, the manufacturers’ protocols were followed.

The homeostatic model assessment for insulin resistance (HOMA-IR) was performed to evaluate impaired insulin sensitivity. It was calculated from fasting insulin and glucose concentration using the formula used by Matthews et al. [60].

HOMA-IR = fasting glucose (mmol/liter) × fasting insulin (mU/liter)/22.5.

### 4.5. Milk RNA Isolation and miRNA Expression Analysis

Total RNA extraction and purification from milk, as well as specific miRNA analysis, were performed as previously described [19]. The small RNA-specific RT and PCR primers used were U6 snRNA (Assay ID: 001973), miR-26a (Assay ID: 000405), miR-27a (Assay ID: 000408), miR-29a (Assay ID: 002112), miR-103 (Assay ID: 000439), miR-200a (Assay ID: 000502), miR-200b (Assay ID: 001800), miR-221 (Assay ID: 000524), and miR-222 (Assay ID: 002276) (Applied Biosystems). The threshold cycle (Ct) was calculated by the instrument’s software (StepOne Software v2.2.2, Applied Biosystems) and miRNA expression was calculated as a percentage of the control group at day 5 of lactation using the 2^−ΔΔCt^ method [61]. Expression of U6 snRNA and β-actin were used as references. Finally, miRNA expression was calculated according to each reference and data are presented as the average of both values. The primers used for the β-actin gene are detailed in Table S1 and were supplied by Sigma.

### 4.6. Tissue RNA Isolation and Gene Expression Analysis

Total RNA extraction was carried out using the EaZy Nucleic Acid Isolation Kit (OMEGA Bio-Tek Inc., Norcross, GA, USA), following the manufacturer’s instructions. RNA was extracted from rWAT, BAT, liver, and MG. Isolated RNA was quantified using the NanoDrop ND-1000 spectrophotometer (NanoDrop Technologies Inc., Wilmington, DE, USA), and mRNA expression of genes related to leptin and adiponectin function, as well as for their relevance in lipid metabolism, were analysed in the collected tissues by Real-time quantitative reverse transcriptase-polymerase chain reaction (qRT-PCR), whose conditions have been previously described by the group [62]. A melting curve was produced in every reaction to verify the purity of the products. The relative expression of each mRNA was calculated as a percentage of the control group following the same procedure described above. GDP dissociation inhibitor (*Gdi*) and *β-actin* were used as reference genes. Each PCR was performed from a diluted cDNA template (1/5), forward and reverse primers (5 μM each), and Power SYBER Green PCR Master Mix (Applied Biosystems, Foster City, CA, USA). All primers were obtained from Sigma Aldrich Química SA (Madrid, Spain) whose sequences are detailed in Table S1.

### 4.7. Statistical Analyses

Data analyses were performed with SPSS v21 for Windows (SPSS, Chicago, IL, United States). For multiple comparisons, a repeated measure analysis of variance (rANOVA) was used to assess the effect of lactation (at days 5, 10, and 15) and diet on L/A ratio and milk miRNA expression. Regarding offspring data, for multiple comparisons, a two-way ANOVA was performed to assess the effect of sex and diet, and a three-way ANOVA for sex, diet, and feeding conditions effects. When the homogeneity of variances was violated, variables were logarithmically transformed. Single comparisons were evaluated by Student’s *t*-test.

Additionally, Spearman’s rank correlation test was conducted to assess the relationship between different parameters. Data are presented as the mean ± Standard Error of the Mean (SEM), and results were considered statistically significant when the *p*-value ≤ 0.05.

## 5. Conclusions

In the current study, we showed how a combination of dietary supplementation with OA, betaine, and Leu during lactation in rats altered milk leptin and adiponectin concentrations, as well as its miRNA profile, concretely, miR-27a, miR-103, miR-200a, and miR-222 levels. Furthermore, the healthy maternal diet ingested during lactation that reduced maternal adiposity effects, caused adverse programming effects in the offspring, particularly increased adiposity, and reduced insulin sensitivity. These results reinforced the importance of ensuring a correct supply of leptin and adiponectin during the perinatal stage, specifically during lactation. Additionally, the results pointed out these miRNAs as potential indicators of the L/A axis in breast milk, which might be proposed as sensitive parameters for the early postnatal programming of the offspring.

## Figures and Tables

**Table 1:** Composition of chow diet and its customized version with the TX combination.

Description	SAFE–Chow Diet (A04)	SAFE–Customized Chow TX Diet
Energy density (kcal/g)	3.33	3.33
Macronutrient composition (%; g/100 g)		
Carbohydrates	60	60
Lipids	3	3
Proteins	16	16
Others (fibre, micronutrients, and water)	21	21
Caloric information (%; Kcal/100 Kcal)		
Carbohydrates	72.5	72.5
Lipids	8.2	8.2
Proteins	19.3	19.3
TX composition (g/kg)		
Oleic acid	4.8	9.6
Leucine	11	22
Betaine	0	1.6

## Data Availability

Not applicable.

## Supplementary Materials

The following supporting information can be downloaded at: https://www.mdpi.com/article/10.3390/ijms23137237/s1.
