# Supplementary material for: Breast Milk MicroRNAs Related to Leptin and Adiponectin Function Can Be Modulated by Maternal Diet and Influence Offspring Phenotype in Rats
Source: Int J Mol Sci. 2022 Jun 29;23(13):7237. doi: 10.3390/ijms23137237 (PMC9266562; doi:10.3390/ijms23137237)
Supplement: Supplementary file 1 [file ijms-23-07237-s001.zip › ijms-1777738-supplementary.pdf]

**Table S1**. Nucleotide sequences of the primers used and amplicon size in base pairs (bp) of the studied genes.

| Gene symbol | Forward primer | Reverse primer | Amplicon size (bp) |
|---|---|---|---|
| *Adipoq* | GCTCAGGATGCTACTGTTG | TCTCACCCTTAGGACCAAG | 241 |
| *Adipor1* | AGCACCGGCAGACAAGAG | GGTGGGTACAACACCACTCA | 62 |
| *Adipor2* | AAAAGGAAAGGCAGAGAATGG | TACACACAGAGACGGGCAAC | 168 |
| *Cpt1a* | GCTCGCACATTACAAGGACAT | TGGACACCACATAGAGGCAG | 250 |
| *Cpt1b* | GCAAACTGGACCGAGAAGAG | CCTTGAAGAAGCGACCTTTG | 180 |
| *Fasn* | AGAGAGAGGCTGTGCTTGC | CTGAGGTCCAGAGATGGTCA | 179 |
| *Gdi* | CCGCACAAGGCAAATACATC | GACTCTCTGAACCGTCATCAA | 159 |
| *Lep* | TTCACACACGCAGTCGGTAT | AGGTCTCGCAGGTTCTCCAG | 186 |
| *Lepr* | AGCCAAACAAAAGCACCATT | TCCTGAGCCATCCAGTCTCT | 174 |
| *Pnpla2* | TCGTATCACCAACACCAGCA | CCCTCCAGTCCTCTCCTCA | 244 |
| *Pparg* | GATCCTCCTGTTGACCCAGA | TCAAAGGAATGGGAGTGGTC | 164 |
| *Scd1* | ATCCCCTCCTCCAAGGTCTA | CGGGCCCATTCATATACATC | 188 |
| *Srebp1* | CCCACCCCCTTACACACC | GCCTGCGGTCTTCATTGT | 198 |
| *Ucp1* | GGGCTGATTCCTTTTGGTCT | GGTGGTGATGGTCCCTAAGA | 229 |
| *β-actin* | TACAGCTTCACCACCACAGC | TCTCCAGGGAGGAAGAGGAT | 120 |

**Table S2**. Associations between leptin and adiponectin concentrations and maternal parameters throughout lactation with the expression of selected miRNAs in milk at day 5 (d5). To study the effect of the TX diet, data was divided in both groups. Statistics: r, Spearman rank coefficient significant at *, p≤0.05; **, p≤0.00.

| | | | **Leptin** | | | | | | | **Adiponectin** | | | | | | **Maternal body weight** | | | | **Maternal body fat** | | | | **Cumulative intake** |
|---|---|---|---|---|---|---|---|---|---|---|---|---|---|---|---|---|---|---|---|---|---|---|---|---|
| | | | **Milk** | | | **Plasma** | | | | **Milk** | | | **Plasma** | | | | | | | | | | | |
| d5 | | | d5 | d10 | d15 | d5 | d10 | d15 | d21 | d5 | d10 | d15 | d5 | d10 | d15 | d5 | d10 | d15 | d21 | d5 | d10 | d15 | d21 | d21 |
| **miR-26a** | Control | r | **-0.857*** | -0.357 | 0.107 | -0.214 | -0.464 | -0.250 | 0.107 | -0.577 | 0.000 | -0.250 | 0.505 | 0.500 | 0.500 | -0.214 | -0.360 | -0.214 | -0.273 | 0.107 | -0.143 | 0.000 | 0.000 | 0.107 |
| | | p | 0.014 | 0.432 | 0.819 | 0.645 | 0.294 | 0.589 | 0.819 | 0.175 | 1.000 | 0.589 | 0.248 | 0.391 | 0.253 | 0.645 | 0.427 | 0.645 | 0.554 | 0.819 | 0.760 | 1.000 | 1.000 | 0.819 |
| | TX | r | 0.714 | 0.607 | 0.679 | **0.986**** | **0.786*** | 0.500 | 0.393 | 0.750 | 0.571 | 0.321 | 0.679 | 0.700 | 0.250 | **0.793*** | **0.857*** | **1.000**** | **0.893**** | 0.393 | 0.214 | 0.286 | 0.321 | 0.750 |
| | | p | 0.071 | 0.148 | 0.094 | 0.000 | 0.036 | 0.253 | 0.383 | 0.052 | 0.180 | 0.482 | 0.094 | 0.188 | 0.589 | 0.033 | 0.014 | | 0.007 | 0.383 | 0.645 | 0.535 | 0.482 | 0.052 |
| **miR-27a** | Control | r | **-0.857*** | -0.250 | 0.143 | -0.214 | -0.429 | -0.250 | 0.286 | -0.378 | 0.071 | -0.107 | 0.595 | 0.600 | 0.500 | -0.179 | -0.234 | -0.179 | -0.018 | 0.143 | -0.071 | 0.250 | 0.250 | 0.107 |
| | | p | 0.014 | 0.589 | 0.760 | 0.645 | 0.337 | 0.589 | 0.535 | 0.403 | 0.879 | 0.819 | 0.159 | 0.285 | 0.253 | 0.702 | 0.613 | 0.702 | 0.969 | 0.760 | 0.879 | 0.589 | 0.589 | 0.819 |
| | TX | r | 0.643 | 0.714 | **0.821*** | **0.812*** | **0.857*** | **0.857*** | 0.607 | **0.857*** | 0.607 | 0.607 | 0.643 | **0.900*** | 0.571 | 0.487 | 0.571 | **0.857*** | **0.857*** | 0.357 | 0.286 | 0.357 | 0.500 | 0.571 |
| | | p | 0.119 | 0.071 | 0.023 | 0.050 | 0.014 | 0.014 | 0.148 | 0.014 | 0.148 | 0.148 | 0.119 | 0.037 | 0.180 | 0.268 | 0.180 | 0.014 | 0.014 | 0.432 | 0.535 | 0.432 | 0.253 | 0.180 |
| **miR-29a** | Control | r | **-0.857*** | -0.286 | -0.107 | -0.214 | -0.393 | -0.286 | 0.000 | -0.559 | -0.214 | -0.500 | 0.414 | 0.600 | 0.357 | -0.107 | -0.234 | -0.107 | -0.164 | 0.214 | -0.071 | 0.000 | 0.000 | 0.107 |
| | | p | 0.014 | 0.535 | 0.819 | 0.645 | 0.383 | 0.535 | 1.000 | 0.192 | 0.645 | 0.253 | 0.355 | 0.285 | 0.432 | 0.819 | 0.613 | 0.819 | 0.726 | 0.645 | 0.879 | 1.000 | 1.000 | 0.819 |
| | TX | r | 0.679 | 0.643 | 0.429 | **0.986**** | 0.571 | 0.179 | 0.107 | 0.571 | 0.321 | 0.000 | 0.357 | 0.700 | -0.036 | **0.811*** | **0.857*** | **0.893**** | 0.714 | 0.071 | -0.107 | 0.036 | 0.000 | 0.714 |
| | | p | 0.094 | 0.119 | 0.337 | 0.000 | 0.180 | 0.702 | 0.819 | 0.180 | 0.482 | 1.000 | 0.432 | 0.188 | 0.939 | 0.027 | 0.014 | 0.007 | 0.071 | 0.879 | 0.819 | 0.939 | 1.000 | 0.071 |
| **miR-103** | Control | r | -0.750 | -0.393 | 0.071 | -0.250 | -0.500 | -0.214 | 0.214 | -0.450 | -0.071 | -0.179 | 0.631 | 0.100 | 0.643 | -0.143 | -0.306 | -0.143 | -0.109 | 0.036 | -0.179 | -0.071 | -0.071 | 0.250 |
| | | p | 0.052 | 0.383 | 0.879 | 0.589 | 0.253 | 0.645 | 0.645 | 0.310 | 0.879 | 0.702 | 0.129 | 0.873 | 0.119 | 0.760 | 0.504 | 0.760 | 0.816 | 0.939 | 0.702 | 0.879 | 0.879 | 0.589 |
| | TX | r | 0.643 | 0.679 | **0.786*** | **0.928**** | 0.750 | 0.643 | 0.357 | **0.857*** | 0.464 | 0.357 | 0.643 | 0.700 | 0.286 | **0.757*** | **0.821*** | **0.964**** | **0.857*** | 0.286 | 0.143 | 0.179 | 0.250 | 0.714 |
| | | p | 0.119 | 0.094 | 0.036 | 0.008 | 0.052 | 0.119 | 0.432 | 0.014 | 0.294 | 0.432 | 0.119 | 0.188 | 0.535 | 0.049 | 0.023 | 0.000 | 0.014 | 0.535 | 0.760 | 0.702 | 0.589 | 0.071 |
| **miR-200a** | Control | r | -0.750 | -0.393 | 0.071 | -0.250 | -0.500 | -0.214 | 0.214 | -0.450 | -0.071 | -0.179 | 0.631 | 0.100 | 0.643 | -0.143 | -0.306 | -0.143 | -0.109 | 0.036 | -0.179 | -0.071 | -0.071 | 0.250 |
| | | p | 0.052 | 0.383 | 0.879 | 0.589 | 0.253 | 0.645 | 0.645 | 0.310 | 0.879 | 0.702 | 0.129 | 0.873 | 0.119 | 0.760 | 0.504 | 0.760 | 0.816 | 0.939 | 0.702 | 0.879 | 0.879 | 0.589 |
| | TX | r | **0.786*** | **0.857*** | 0.607 | **0.812*** | **0.857*** | 0.429 | 0.500 | 0.714 | 0.679 | 0.429 | 0.357 | **0.900*** | 0.393 | 0.468 | 0.571 | **0.857*** | **0.964**** | 0.143 | 0.000 | 0.214 | 0.429 | 0.679 |
| | | p | 0.036 | 0.014 | 0.148 | 0.050 | 0.014 | 0.337 | 0.253 | 0.071 | 0.094 | 0.337 | 0.432 | 0.037 | 0.383 | 0.289 | 0.180 | 0.014 | 0.000 | 0.760 | 1.000 | 0.645 | 0.337 | 0.094 |
| **miR-200b** | Control | r | -0.714 | -0.250 | 0.036 | -0.179 | -0.393 | -0.143 | 0.286 | -0.306 | -0.214 | -0.214 | 0.667 | 0.100 | 0.607 | 0.000 | -0.126 | 0.000 | 0.073 | 0.179 | -0.036 | 0.071 | 0.071 | 0.286 |
| | | p | 0.071 | 0.589 | 0.939 | 0.702 | 0.383 | 0.760 | 0.535 | 0.504 | 0.645 | 0.645 | 0.102 | 0.873 | 0.148 | 1.000 | 0.788 | 1.000 | 0.877 | 0.702 | 0.939 | 0.879 | 0.879 | 0.535 |
| | TX | r | 0.714 | 0.643 | 0.643 | **0.899*** | 0.643 | 0.286 | 0.179 | 0.714 | 0.357 | 0.036 | 0.429 | 0.600 | -0.036 | **0.811*** | **0.857*** | **0.929**** | **0.821*** | 0.071 | -0.143 | 0.000 | 0.071 | **0.893**** |
| | | p | 0.071 | 0.119 | 0.119 | 0.015 | 0.119 | 0.535 | 0.702 | 0.071 | 0.432 | 0.939 | 0.337 | 0.285 | 0.939 | 0.027 | 0.014 | 0.003 | 0.023 | 0.879 | 0.760 | 1.000 | 0.879 | 0.007 |
| **miR-221** | Control | r | -0.214 | -0.321 | **-0.786*** | -0.429 | -0.357 | -0.393 | -0.036 | -0.018 | -0.536 | -0.714 | 0.270 | -0.100 | 0.214 | 0.179 | 0.126 | 0.179 | 0.564 | -0.143 | -0.286 | -0.357 | -0.357 | 0.429 |
| | | p | 0.645 | 0.482 | 0.036 | 0.337 | 0.432 | 0.383 | 0.939 | 0.969 | 0.215 | 0.071 | 0.558 | 0.873 | 0.645 | 0.702 | 0.788 | 0.702 | 0.187 | 0.760 | 0.535 | 0.432 | 0.432 | 0.337 |
| | TX | r | **0.929**** | 0.429 | 0.107 | 0.696 | **0.786*** | 0.214 | **0.786*** | 0.071 | 0.714 | 0.179 | 0.143 | 0.200 | 0.321 | 0.054 | 0.107 | 0.500 | 0.536 | 0.214 | 0.143 | 0.679 | 0.679 | 0.571 |
| | | p | 0.003 | 0.337 | 0.819 | 0.125 | 0.036 | 0.645 | 0.036 | 0.879 | 0.071 | 0.702 | 0.760 | 0.747 | 0.482 | 0.908 | 0.819 | 0.253 | 0.215 | 0.645 | 0.760 | 0.094 | 0.094 | 0.180 |
| **miR-222** | Control | r | -0.714 | -0.429 | 0.179 | -0.179 | -0.536 | -0.143 | 0.321 | -0.414 | 0.000 | -0.214 | 0.703 | 0.100 | 0.750 | -0.071 | -0.270 | -0.071 | -0.164 | 0.000 | -0.250 | -0.107 | -0.107 | 0.393 |
| | | p | 0.071 | 0.337 | 0.702 | 0.702 | 0.215 | 0.760 | 0.482 | 0.355 | 1.000 | 0.645 | 0.078 | 0.873 | 0.052 | 0.879 | 0.558 | 0.879 | 0.726 | 1.000 | 0.589 | 0.819 | 0.819 | 0.383 |
| | TX | r | 0.679 | 0.393 | 0.679 | 0.754 | 0.643 | 0.321 | 0.357 | 0.607 | 0.429 | 0.071 | 0.464 | 0.300 | 0.000 | 0.577 | 0.607 | 0.750 | 0.750 | 0.179 | -0.036 | 0.143 | 0.286 | **0.929**** |
| | | p | 0.094 | 0.383 | 0.094 | 0.084 | 0.119 | 0.482 | 0.432 | 0.148 | 0.337 | 0.879 | 0.294 | 0.624 | 1.000 | 0.175 | 0.148 | 0.052 | 0.052 | 0.702 | 0.939 | 0.760 | 0.535 | 0.003 |

**Table S3**. Associations between leptin and adiponectin concentrations and maternal parameters throughout lactation with the expression of selected miRNAs in milk at day 15 (d15). To study the effect of the TX diet, data was divided in both groups. Statistics: r, Spearman rank coefficient significant at *, p≤0.05; **, p≤0.00.

| | | | **Leptin** | | | | | | | **Adiponectin** | | | | | | **Maternal body weight** | | | | **Maternal body fat** | | | | **Cumulative intake** |
|---|---|---|---|---|---|---|---|---|---|---|---|---|---|---|---|---|---|---|---|---|---|---|---|---|
| | | | **Milk** | | | **Plasma** | | | | **Milk** | | | **Plasma** | | | | | | | | | | | |
| d15 | | | d5 | d10 | d15 | d5 | d10 | d15 | d21 | d5 | d10 | d15 | d5 | d10 | d15 | d5 | d10 | d15 | d21 | d5 | d10 | d15 | d21 | d21 |
| **miR-26a** | Control | r | 0.036 | -0.214 | -0.714 | -0.429 | -0.143 | -0.286 | -0.321 | -0.090 | -0.607 | -0.214 | 0.018 | -0.800 | 0.036 | -0.036 | -0.090 | -0.036 | 0.382 | -0.143 | -0.071 | -0.464 | -0.464 | 0.036 |
| | | p | 0.939 | 0.645 | 0.071 | 0.337 | 0.760 | 0.535 | 0.482 | 0.848 | 0.148 | 0.645 | 0.969 | 0.104 | 0.939 | 0.939 | 0.848 | 0.939 | 0.398 | 0.760 | 0.879 | 0.294 | 0.294 | 0.939 |
| | TX | r | 0.179 | 0.500 | 0.000 | 0.058 | -0.071 | -0.321 | -0.250 | 0.071 | -0.250 | -0.357 | -0.714 | 0.100 | -0.321 | -0.162 | -0.143 | -0.107 | 0.071 | **-0.857*** | **-0.893**** | -0.607 | -0.357 | 0.286 |
| | | p | 0.702 | 0.253 | 1.000 | 0.913 | 0.879 | 0.482 | 0.589 | 0.879 | 0.589 | 0.432 | 0.071 | 0.873 | 0.482 | 0.728 | 0.760 | 0.819 | 0.879 | 0.014 | 0.007 | 0.148 | 0.432 | 0.535 |
| **miR-27a** | Control | r | 0.107 | 0.179 | -0.571 | -0.036 | 0.143 | 0.036 | 0.000 | 0.288 | **-0.821*** | -0.464 | 0.144 | -0.300 | 0.000 | 0.464 | 0.468 | 0.464 | 0.655 | 0.286 | 0.250 | -0.036 | -0.036 | 0.286 |
| | | p | 0.819 | 0.702 | 0.180 | 0.939 | 0.760 | 0.939 | 1.000 | 0.531 | 0.023 | 0.294 | 0.758 | 0.624 | 1.000 | 0.294 | 0.289 | 0.294 | 0.111 | 0.535 | 0.589 | 0.939 | 0.939 | 0.535 |
| | TX | r | 0.750 | 0.607 | 0.036 | **0.899*** | 0.429 | -0.179 | 0.071 | 0.179 | 0.214 | -0.286 | -0.107 | 0.200 | -0.214 | 0.505 | 0.536 | 0.607 | 0.464 | -0.250 | -0.393 | 0.000 | -0.071 | 0.643 |
| | | p | 0.052 | 0.148 | 0.939 | 0.015 | 0.337 | 0.702 | 0.879 | 0.702 | 0.645 | 0.535 | 0.819 | 0.747 | 0.645 | 0.248 | 0.215 | 0.148 | 0.294 | 0.589 | 0.383 | 1.000 | 0.879 | 0.119 |
| **miR-29a** | Control | r | -0.071 | -0.286 | -0.643 | -0.393 | -0.214 | -0.250 | -0.286 | -0.198 | -0.643 | -0.321 | 0.126 | -0.800 | 0.179 | 0.000 | -0.126 | 0.000 | 0.255 | -0.107 | -0.107 | -0.536 | -0.536 | 0.143 |
| | | p | 0.879 | 0.535 | 0.119 | 0.383 | 0.645 | 0.589 | 0.535 | 0.670 | 0.119 | 0.482 | 0.788 | 0.104 | 0.702 | 1.000 | 0.788 | 1.000 | 0.582 | 0.819 | 0.819 | 0.215 | 0.215 | 0.760 |
| | TX | r | 0.679 | 0.643 | 0.393 | 0.754 | 0.393 | 0.107 | 0.000 | 0.464 | 0.000 | -0.321 | -0.036 | 0.100 | -0.286 | 0.595 | 0.607 | 0.643 | 0.464 | -0.393 | -0.536 | -0.214 | -0.214 | **0.821*** |
| | | p | 0.094 | 0.119 | 0.383 | 0.084 | 0.383 | 0.819 | 1.000 | 0.294 | 1.000 | 0.482 | 0.939 | 0.873 | 0.535 | 0.159 | 0.148 | 0.119 | 0.294 | 0.383 | 0.215 | 0.645 | 0.645 | 0.023 |
| **miR-103** | Control | r | 0.107 | 0.000 | -0.643 | -0.286 | 0.071 | -0.179 | -0.393 | -0.036 | -0.643 | -0.143 | -0.144 | -0.800 | -0.179 | 0.000 | 0.000 | 0.000 | 0.346 | 0.036 | 0.143 | -0.286 | -0.286 | -0.143 |
| | | p | 0.819 | 1.000 | 0.119 | 0.535 | 0.879 | 0.702 | 0.383 | 0.939 | 0.119 | 0.760 | 0.758 | 0.104 | 0.702 | 1.000 | 1.000 | 1.000 | 0.448 | 0.939 | 0.760 | 0.535 | 0.535 | 0.760 |
| | TX | r | 0.536 | 0.679 | 0.321 | 0.754 | 0.250 | 0.036 | -0.143 | 0.429 | -0.143 | -0.357 | -0.214 | 0.100 | -0.321 | 0.487 | 0.500 | 0.500 | 0.357 | -0.571 | -0.679 | -0.393 | -0.357 | 0.679 |
| | | p | 0.215 | 0.094 | 0.482 | 0.084 | 0.589 | 0.939 | 0.760 | 0.337 | 0.760 | 0.432 | 0.645 | 0.873 | 0.482 | 0.268 | 0.253 | 0.253 | 0.432 | 0.180 | 0.094 | 0.383 | 0.432 | 0.094 |
| **miR-200a** | Control | r | 0.071 | -0.429 | -0.714 | -0.607 | -0.286 | -0.464 | -0.500 | -0.270 | -0.286 | -0.036 | -0.162 | **-0.900*** | -0.036 | -0.357 | -0.414 | -0.357 | 0.145 | -0.464 | -0.321 | -0.679 | -0.679 | -0.143 |
| | | p | 0.879 | 0.337 | 0.071 | 0.148 | 0.535 | 0.294 | 0.253 | 0.558 | 0.535 | 0.939 | 0.728 | 0.037 | 0.939 | 0.432 | 0.355 | 0.432 | 0.756 | 0.294 | 0.482 | 0.094 | 0.094 | 0.760 |
| | TX | r | 0.321 | 0.679 | 0.357 | 0.348 | 0.107 | 0.107 | -0.179 | 0.429 | -0.286 | -0.286 | -0.429 | 0.100 | -0.250 | 0.162 | 0.179 | 0.214 | 0.214 | **-0.786*** | **-0.821*** | -0.571 | -0.393 | 0.500 |
| | | p | 0.482 | 0.094 | 0.432 | 0.499 | 0.819 | 0.819 | 0.702 | 0.337 | 0.535 | 0.535 | 0.337 | 0.873 | 0.589 | 0.728 | 0.702 | 0.645 | 0.645 | 0.036 | 0.023 | 0.180 | 0.383 | 0.253 |
| **miR-200b** | Control | r | 0.036 | -0.214 | -0.714 | -0.429 | -0.143 | -0.286 | -0.321 | -0.090 | -0.607 | -0.214 | 0.018 | -0.800 | 0.036 | -0.036 | -0.090 | -0.036 | 0.382 | -0.143 | -0.071 | -0.464 | -0.464 | 0.036 |
| | | p | 0.939 | 0.645 | 0.071 | 0.337 | 0.760 | 0.535 | 0.482 | 0.848 | 0.148 | 0.645 | 0.969 | 0.104 | 0.939 | 0.939 | 0.848 | 0.939 | 0.398 | 0.760 | 0.879 | 0.294 | 0.294 | 0.939 |
| | TX | r | 0.464 | 0.571 | -0.107 | 0.754 | 0.143 | -0.179 | -0.143 | 0.071 | -0.107 | -0.357 | -0.357 | -0.100 | -0.250 | 0.306 | 0.321 | 0.321 | 0.143 | -0.500 | -0.536 | -0.214 | -0.321 | 0.321 |
| | | p | 0.294 | 0.180 | 0.819 | 0.084 | 0.760 | 0.702 | 0.760 | 0.879 | 0.819 | 0.432 | 0.432 | 0.873 | 0.589 | 0.504 | 0.482 | 0.482 | 0.760 | 0.253 | 0.215 | 0.645 | 0.482 | 0.482 |
| **miR-221** | Control | r | 0.771 | 0.600 | 0.086 | 0.771 | 0.600 | **0.829*** | -0.029 | 0.493 | -0.657 | -0.143 | -0.319 | -0.600 | -0.257 | **0.943**** | **0.841*** | **0.943**** | -0.029 | 0.600 | 0.543 | -0.086 | -0.086 | 0.314 |
| | | p | 0.072 | 0.208 | 0.872 | 0.072 | 0.208 | 0.042 | 0.957 | 0.321 | 0.156 | 0.787 | 0.538 | 0.400 | 0.623 | 0.005 | 0.036 | 0.005 | 0.956 | 0.208 | 0.266 | 0.872 | 0.872 | 0.544 |
| | TX | r | 0.250 | -0.036 | -0.357 | 0.145 | 0.000 | -0.036 | 0.071 | -0.321 | -0.107 | -0.286 | -0.107 | -0.700 | -0.107 | 0.072 | 0.036 | 0.036 | -0.286 | -0.036 | 0.036 | 0.286 | -0.071 | -0.036 |
| | | p | 0.589 | 0.939 | 0.432 | 0.784 | 1.000 | 0.939 | 0.879 | 0.482 | 0.819 | 0.535 | 0.819 | 0.188 | 0.819 | 0.878 | 0.939 | 0.939 | 0.535 | 0.939 | 0.939 | 0.535 | 0.879 | 0.939 |
| **miR-222** | Control | r | 0.143 | -0.214 | -0.429 | -0.214 | 0.000 | -0.143 | -0.679 | -0.432 | -0.393 | -0.179 | -0.396 | -0.600 | -0.214 | -0.214 | -0.360 | -0.214 | -0.364 | -0.143 | -0.071 | -0.679 | -0.679 | -0.250 |
| | | p | 0.760 | 0.645 | 0.337 | 0.645 | 1.000 | 0.760 | 0.094 | 0.333 | 0.383 | 0.702 | 0.379 | 0.285 | 0.645 | 0.645 | 0.427 | 0.645 | 0.423 | 0.760 | 0.879 | 0.094 | 0.094 | 0.589 |
| | TX | r | **0.786*** | **0.857*** | 0.571 | 0.580 | 0.679 | 0.357 | 0.393 | 0.607 | 0.357 | 0.107 | -0.036 | 0.300 | 0.143 | 0.252 | 0.321 | 0.607 | 0.714 | -0.321 | -0.429 | -0.036 | 0.214 | **0.786*** |
| | | p | 0.036 | 0.014 | 0.180 | 0.228 | 0.094 | 0.432 | 0.383 | 0.148 | 0.432 | 0.819 | 0.939 | 0.624 | 0.760 | 0.585 | 0.482 | 0.148 | 0.071 | 0.482 | 0.337 | 0.939 | 0.645 | 0.036 |

**Table S4**. Associations between milk L/A ratio and diet-modulated miRNAs throughout lactation with offspring body fat in adulthood divided by sex. Statistics: r, Spearman rank coefficient significant at *, p≤0.05; **, p≤0.00.

| | | | Male offspring body fat (%) | | Female offspring body fat (%) | |
|---|---|---|---|---|---|---|
| Milk | | | d60 | d90 | d60 | d90 |
| **L/A ratio** | d5 | r | 0.299 | 0.262 | 0.266 | 0.422 |
| | | p | 0.299 | 0.366 | 0.358 | 0.132 |
| | d10 | r | 0.073 | 0.020 | 0.373 | 0.148 |
| | | p | 0.805 | 0.946 | 0.189 | 0.614 |
| | d15 | r | 0.367 | 0.486 | **0.741**** | **0.556*** |
| | | p | 0.197 | 0.078 | 0.002 | 0.039 |
| **miR-27a** | d5 | r | 0.068 | -0.275 | 0.002 | 0.015 |
| | | p | 0.817 | 0.342 | 0.994 | 0.958 |
| | d15 | r | -0.429 | **-0.741**** | **-0.560*** | **-0.662*** |
| | | p | 0.126 | 0.002 | 0.037 | 0.010 |
| **miR-103** | d5 | r | -0.002 | -0.332 | 0.046 | -0.020 |
| | | p | 0.994 | 0.246 | 0.876 | 0.946 |
| | d15 | r | -0.174 | **-0.657*** | -0.165 | **-0.525*** |
| | | p | 0.553 | 0.011 | 0.573 | 0.054 |
| **miR-200a** | d5 | r | -0.169 | -0.407 | -0.055 | -0.204 |
| | | p | 0.563 | 0.149 | 0.852 | 0.483 |
| | d15 | r | -0.103 | **-0.596*** | -0.011 | **-0.609*** |
| | | p | 0.725 | 0.025 | 0.970 | 0.021 |
| **miR-222** | d5 | r | -0.266 | **-0.534*** | -0.007 | -0.253 |
| | | p | 0.358 | 0.049 | 0.982 | 0.383 |
| | d15 | r | -0.257 | **-0.613*** | -0.059 | **-0.582*** |
| | | p | 0.375 | 0.020 | 0.840 | 0.029 |
